# *Ancylostoma* in dogs in the Caribbean: a review and study from St. Kitts, West Indies

**DOI:** 10.1186/s13071-022-05254-2

**Published:** 2022-04-21

**Authors:** Jenny Kim, Araceli Lucio-Forster, Jennifer K. Ketzis

**Affiliations:** 1 Department of Biomedical Sciences, Ross University School of Veterinary Medicine, Basseterre, Saint Kitts and Nevis; 2 Department of Microbiology and Immunology, Cornell University College of Veterinary Medicine, Ithaca, NY USA

**Keywords:** Canine, *Ancylostoma caninum*, *Ancylostoma braziliense*, Hookworm, Helminths, Zoonosis

## Abstract

**Background:**

Little is known about the prevalence of *Ancylostoma* in dogs in the Caribbean. In view of the number of owned free-roaming and feral dogs within the islands and the ideal subtropical climate for parasite development and environmental survival, *Ancylostoma* could pose a threat to the health of the dogs as well as a zoonotic risk to people.

**Methods:**

To determine whether generalities about *Ancylostoma* in dogs in the Caribbean could be made and to obtain a better understanding of the prevalence, published (Scielo, Scopus, and PubMed databases) and gray (e.g., student theses, conference presentations) literature was reviewed. Retrieved manuscripts were screened, and relevant data (year, location, dog population, method of diagnosis, positivity rate) were extracted. Data from two dog populations on St. Kitts also were included: a 2014 field study involving dogs with limited veterinary care and data from the Ross University School of Veterinary Medicine’s Veterinary Clinic records for 2018–2019.

**Results:**

Fourteen manuscripts from the 1950s to 2019, representing ten of the Caribbean islands/countries and the Bahamas, were identified. Methods of diagnosing infection status ranged from simple qualitative or quantitative flotation methods to centrifugation with Sheather’s sugar flotation solution or necropsy. Dog populations sampled included stray, owned free-roaming, and owned confined. Reported rates of *Ancylostoma* infection ranged from 10 to 91%. Studies from the last 10 years indicate positivity rates of 21 to 73%. *Ancylostoma* positivity rates in the St. Kitts’ populations were 61% and 10% for the 2014 and 2018–2019 populations, respectively.

**Conclusions:**

There was no indication that hookworm prevalence has changed over time in the Caribbean, and there were no obvious differences between owned and unowned dogs or free-roaming and confined dogs. The data from St. Kitts were on par with positivity rates from the other islands within the last 10 years and reflective of the impact that veterinary care, including anthelmintic treatment, is expected to have on parasites in pets. There is a clear need to expand the available data for the region and improve control programs for *Ancylostoma* infections to protect both canine and human health.

**Graphical Abstract:**

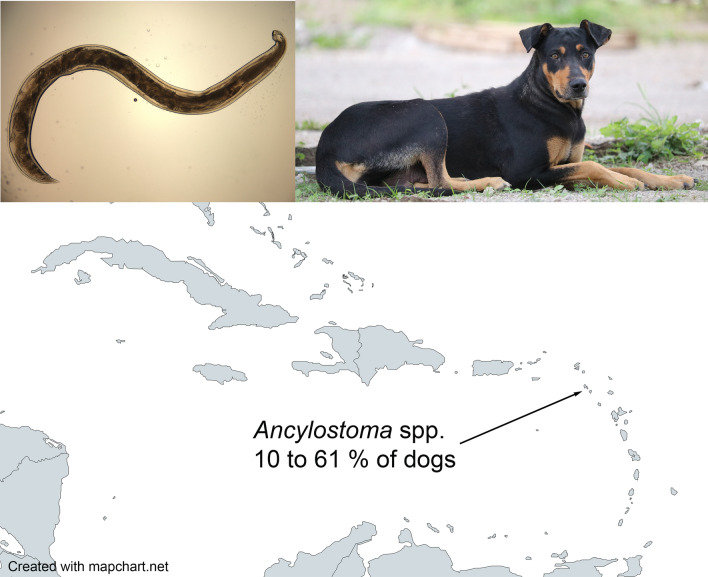

**Supplementary Information:**

The online version contains supplementary material available at 10.1186/s13071-022-05254-2.

## Background

Understanding the prevalence of *Ancylostoma* in dogs within the Caribbean islands is critical for the development of effective control programs to protect canine and human health, especially in light of concerns with developing anthelmintic resistance [1–3]. *Ancylostoma caninum* and, to a lesser extent and not well documented, *Ancylostoma braziliense* occur in the Caribbean with both parasites capable of infecting dogs and causing zoonotic disease (e.g., cutaneous larval migrans) in people [3–5]. The latter species also uses cats as a definitive host. Canine infection occurs when third-stage larvae enter dogs orally (from the environment, in prey, or, additionally for *A. caninum*, via milk during nursing) or percutaneously. Human infection occurs primarily via the percutaneous route.

Little is known about the prevalence of *Ancylostoma*, and hence the risk to the canine and human populations, within the Caribbean islands. Assessing prevalence is challenging as many interrelated factors influence infection rates in populations including how owned dogs are managed (free-roaming or confined), financial and physical access to veterinary care, frequency of use of anthelmintics, level of feral dog populations, and removal of feces from the environment. Further confounding the problem is the high variability in the sensitivity of diagnostic methods available to detect infections [6, 7]; hence, extrapolation from one study to a broader population is difficult.

To determine whether generalities relative to the positivity rates of *Ancylostoma* in dogs in the Caribbean could be identified, a review of the literature was conducted, and data from two studies on St. Kitts (a Leeward Island in the Lesser Antilles) were examined.

## Methods

### Literature review

A scoping review was performed to characterize and summarize the available information on *Ancylostoma* in dogs in the Caribbean region. In November 2019, Scielo (Scientific Electronic Library Online), Scopus, and PubMed were searched using combinations of the terms “canine,” “dog,” “*Ancylostoma*,” and “hookworm” plus the name of each Caribbean island, “Leeward Island,” “Antilles," “West Indies,” and “Caribbean.” If there was no information found for an island, the search terms “parasite” plus the island name were used. For each search result obtained, the abstract was reviewed to determine relevance. Articles were excluded if the country or the hookworm species was not relevant. In addition to the databases, gray literature from university theses from the region and conference proceedings were searched for data on canine *Ancylostoma* infections. Data gathered from each article included country/island of the study, year of the study, percent of *Ancylostoma* positive dogs, dog population (age and management), and diagnostic method.

### Case study: St. Kitts

Two sets of data were used to assess *Ancylostoma* positivity rate in St. Kitts. The first data set was collected during a field study conducted in 2014 under a Ross University School of Veterinary Medicine (RUSVM) Institutional Animal Care and Use Committee approved protocol (14–3-008). The study population consisted of dogs that did not belong to RUSVM employees or students and represented dogs with limited access to veterinary care. The target enrollment was 90 to 108 dogs, with approximately 10 to 12 from each of the 9 Parishes on St. Kitts. To enroll dogs in the study, researchers visited communities in each Parish, explained the purpose of the work, and obtained owner consent prior to any data or sample collection. Data collection included: age and sex of the dog, anthelmintic treatment history, and general observations on the management system (free-roaming, confined in a fenced area, or tethered). Freshly defecated feces were collected from the ground, placed in a cooler, and then transported to the RUSVM diagnostic laboratory. Samples were stored at 4 to 8 °C until analysis, which was conducted within 3 days of collection. A double centrifugation method with Sheather’s sugar [specific gravity (spg) 1.27–1.28; 59 samples] or zinc sulfate (spg 1.18–1.2; 38 samples) flotation solution was used for the analysis [6]. Briefly, 1 to 2 g feces was weighed, mixed with water, strained, and centrifuged at approximately 500 g for 5 min. The supernatant was poured off and the flotation solution added to the pellet. After mixing, the test tube was filled with the flotation solution to form a positive meniscus. A coverslip was placed on the tube, and the tube was centrifuged once more at 500 g for 5 min. The tube was allowed to sit for a minimum of 10 min before the coverslip was removed and examined at 100 × magnification. All eggs seen were counted and recorded. The number of eggs per gram (epg) of feces in each sample was calculated using the number of eggs seen and the weight of feces used.

The second data set was obtained from the RUSVM Veterinary Clinic database (AVImark®, Covetrus, WI, USA) under an Exemption Certification (19–05-EX) from the RUSVM Institutional Review Board. Fecal examination results for dogs previously tested at the clinic were obtained from AVImark®, the RUSVM clinic database. The database was searched to identify all dogs having had a fecal examination performed between 1 January 2018 and 31 December 2019. Data collected from AVImark® included: age and sex of the dog, date of fecal examination, results, and historical anthelmintic use. For patients with multiple fecal examinations, the most recent fecal examination result was included in the data set to represent infection in a “well-cared for” population. The first fecal examination, potentially from shortly after adoption, also was recorded and considered separately.

For this second data set, feces were analyzed by the RUSVM diagnostic laboratory following a standard double centrifugation procedure using Sheather’s sugar flotation solution (spg 1.27–1.28) with the exception of three samples analyzed with zinc sulfate (spg 1.18–1.2). The procedure differed from that used in the 2014 study in two main respects: first, a 10-min delay before reading the coverslip was not standardized; second, results were recorded as either estimated levels or as positive or negative versus as epg.

For both data sets, ages were categorized as < 6 months old, 6 months to 12 months old, and > 12 months old at the time of the fecal examination, and arithmetic mean positivity rates for each age group and total were calculated. Sex was recorded as male or female without differentiation for spay or neuter status. Anthelmintics reportedly used were reviewed (using product labels and the US Food and Drug Administration freedom of information summaries) for approved treatment for *Ancylostoma* and categorized as monthly treatments (*Dirofilaria immitis* preventative with activity against *Ancylostoma* on the label), other (anthelmintic administered within the previous 6 months), and none (treatment > 6 months ago or no record of anthelmintic purchase). A chi-square or Fisher exact test, when expected frequencies were five or less (vassarstats.net), was used to compare the positivity rate between the two data sets and assess differences based on age, gender, and anthelmintic use. A *P*-value < 0.05 was considered significant.

## Results

### Literature review

**Fig. 1:**
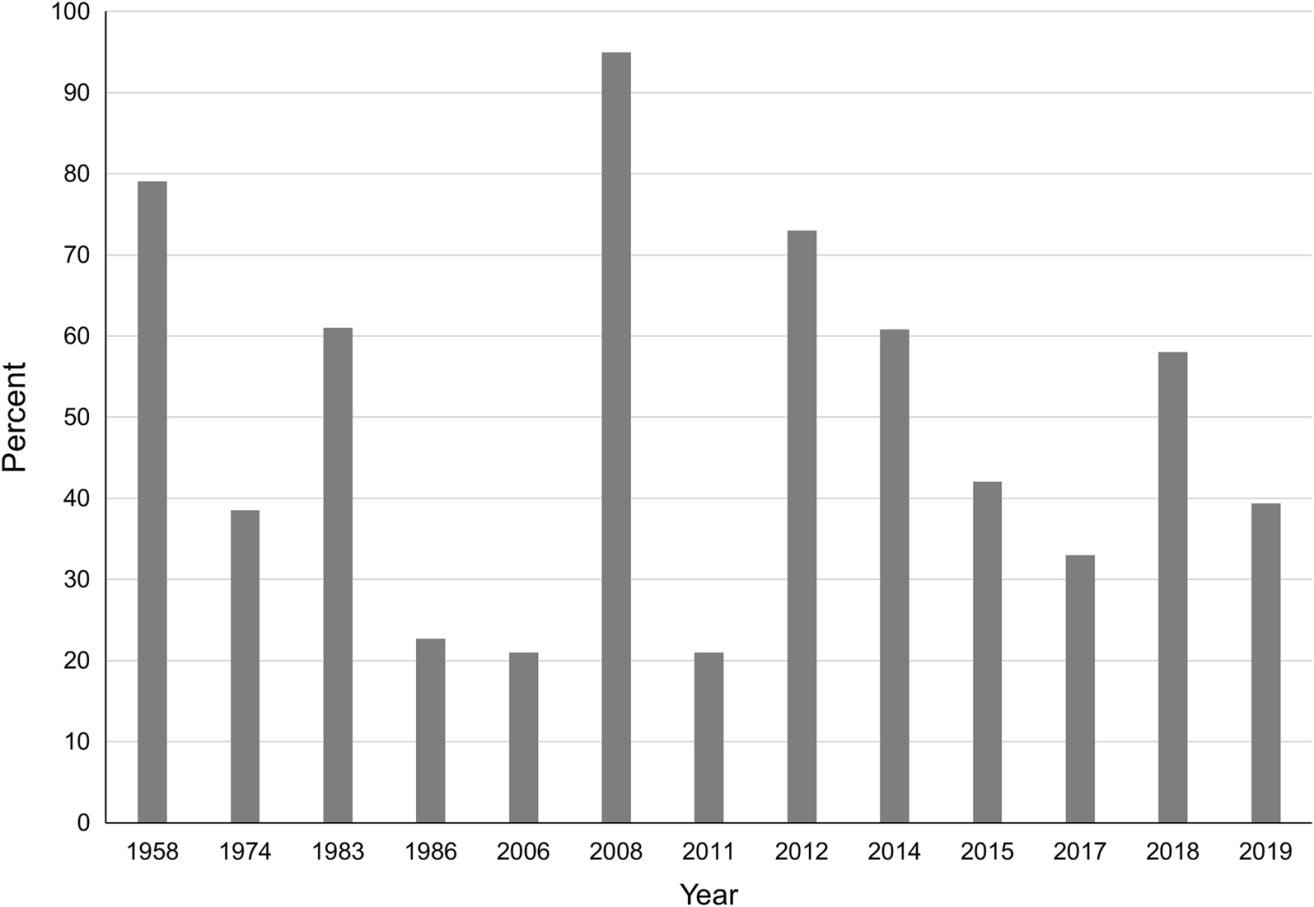
*Ancylostoma* positivity rate by year in the Caribbean. In years with more than one study, data were averaged. Year of end of study or year of study publication was used. Data are based on 11 islands and/or countries within the region

Fourteen relevant articles, five of which were obtained from gray literature, were identified from the 9728 search results recovered through the scoping review. The data gathered from these articles are presented in Table 1. Data from 11 Caribbean islands/countries were available, all with just one data point except for Cuba, Curaçao, and Grenada. Six studies used necropsy for parasite identification with the remainder using fecal-based methods. The *Ancylostoma* positivity rate ranged from 10 to 95% with the lowest and highest positivity rates from studies with the smallest sample sizes (10 and 20 dogs, respectively). Those that used necropsy and larger sample sizes (> 20) had a positivity range of 18.5 to 90.7% while those that used simple flotation or sedimentation methods ranged from 22.7 to 73.0%. Studies conducted over 40 years ago (pre-1980) had a positivity rate of 10 to 90.7%, while those reporting data generated only within the last 10 years ranged from 15.4 to 68.8%. There was no clear trend in positivity rate based on year of the study (Fig. 1), dog ownership status, or age of dog.

**Table 1:** Summary of *Ancylostoma* fecal positivity rate in dogs from studies in the Caribbean islands

Country/Island	Year^a^	Percent positive for *Ancylostoma* spp.^b^		Description	Analysis method	Reference
Aruba	1974	11.1 *A. braziliense* 18.5 *A. caninum*		27 dogs^c^	Necropsy	[23]
Bahamas	1957–1958	50.0	1–6 months	127 dogs^c^ 36 dogs: 1 to 6 mo New Providence and Grand Bahama	Necropsy	[24]
		84.3	Adult			
Bonaire	1974	10.0 *A. caninum*		10 dogs^c^	Necropsy	[23]
Cuba	2005–2006	21.0	Overall	461 stray dogs 248 dogs: < 1 year of age 213 dogs: > 1 year of age Havana	Necropsy	[25]
		14.5	< 1 year			
		26.6	> 1 year			
	2015	39.0	Urban	49 urban owned dogs 59 rural owned dogs No age difference seen Municipality Santa Clara	Preserved 10% phenol Centrifugation with Sheather's sugar	[26]
		40.0	Rural			
	2015	44.6		547 samples from the ground Municipality Camagüey	Flotation in duplicate	[20]
Curaçao	1974	90.7 *A. caninum*		54 dogs^c^	Necropsy	[23]
	2011	21.0	Overall	157 dogs 6 months to 17 years of age 26 from a shelter, remainder owned and examined at a veterinary clinic	Centrifugation with sugar flotation solution	[21]
		22.1	Owned			
		15.4	Shelter			
Dominican Republic	2018	58.0		113 dogs Primarily free-roaming adult dogs San Cristobal	McMaster; saturated salt solution	[27]
Grenada	2007–2008	95.0		20 dogs Free roaming	Double centrifugation; zinc sulfate spg 1.18–1.2	[28]
	2008–2012	73.0	Overall	445 stray dogs Age estimated 8 dogs: < 12 weeks of age 32 dogs: 12 to 24 weeks of age 405 dogs: adult	Stored 10% formalin Flotation; saturated NaCl	[29]
		87.5	< 12 weeks			
		52.2	12–24 wk			
		74.1	Adult			
	2019	68.8		154 dogs Presenting for laboratories at St. George’s University, School of Veterinary Medicine	FecalDx® (fecal antigen), IDEXX Laboratories	[30]
Guadeloupe	1982–1983	86.0		44 dogs^c^	Necropsy and fecal examination (method not described)	[31]
Jamaica	1986	22.7		141 dogs Primarily adult and owned	Ritchie formol-ether in duplicate	[32]
St. Lucia	1983	36.0		22 samples from the ground Samples from free-roaming and non-free-roaming dogs	Modified Ritchie formol-ether	[33]
Trinidad	2017	33.0		100 owned dogs	Simple flotation; sodium nitrate	[22]

^a^Year of study; if study year was not specified, year of publication

^b^Species of *Ancylostoma* indicated only if the study specified the species found

^c^Ownership not specified

### Case study: St. Kitts

Feces from a total of 97 dogs were obtained in the 2014 field study. The overall positivity rate of *Ancylostoma* was found to be 60.8% (59 of 97) (Table 2; Additional file 1). The *Ancylostoma* positivity rate increased with age, although the sample size was small for dogs ≤ 12 months of age. The majority of the dogs (57 of 97; 58.8%) had not received any anthelmintic treatment within the previous 6 months, although nine of these had been treated either as a puppy or > 1 year ago. Only 5.2% (five of 97) were on a monthly heartworm disease preventative. The remaining dogs (35 of 97; 36.1%) had been administered an anthelmintic at least once in the previous 6 months; products used varied from those approved for use in cattle (containing albendazole, fenbendazole or ivermectin) to ones approved for use in dogs (containing pyrantel and/or febantel). Positivity rate decreased in relation to reported anthelmintic use. Most of the dogs were either confined in a fenced area or tethered. However, some were allowed to roam free: 17.0% (10 of 59) of the *Ancylostoma*-positive dogs were free-roaming and 5.3% (2 of 38) of the *Ancylostoma* negatives were free-roaming.

**Table 2:** Positivity rate of *Ancylostoma* in two sets of dogs on St. Kitts

	Field Study^a^ 2014			Clinic Data^a^ 2018–2019			*P*-value^d^
	N (%)	Positive (%)	*P*-value^c^	N (%)	Positive (%)	*P*-value^c^	
Age (months)							
< 6	4 (4.1)	1 (25.0)	0.3899	52 (22.4)	5 (9.6)	0.2924	0.0002
6–12	17 (17.5)	10 (58.8)		27 (11.6)	5 (18.5)		
> 12	76 (78.4)	48 (63.2)		153 (65.9)	13 (8.5)		
Sex							
Male	45 (46.4)	30 (66.7)	0.3741	108 (46.8)	12 (11.1)	0.7401	1.0
Female	52 (53.6)	29 (55.8)		123 (53.2)	11 (8.9)		
Anthelmintic use^b^							
Monthly	5 (5.2)	1 (20.0)	0.0299	121 (52.2)	6 (5.0)	0.0154	0.0001
Other	35 (36.1)	18 (51.4)		36 (15.5)	4 (11.1)		
None	57 (58.8)	40 (70.2)		75 (32.3)	13 (17.3)		
Total	97	59 (60.8)		232	23 (9.9)		0.0001

^a^The 2014 data represent dogs with limited veterinary care. Clinic data, January 2018–December 2019, represent dogs with greater veterinary care

^b^Monthly defined as a *Dirofilaria immitis* preventative excluding Revolution® (selamectin). Other defined as an anthelmintic administered within the previous 6 months with activity against *Ancylostoma*. None indicates the owner had not administered any anthelmintic in the previous 6 months (2014 data) or there were no records of dispensing an anthelmintic (2018–2019 data)

^c^Based on a chi-square or Fisher’s exact test

^d^Based on a *χ*^2^ or Fisher’s exact test; comparison between the two study groups

The 2018–2019 RUSVM clinic database contained fecal examination information for 232 dogs, mainly representative of student-owned dogs from St. Kitts and the university’s teaching dog colony with all of these dogs from St. Kitts. Forty of the 232 dogs had more than one sample analyzed; these fecal examinations were often after recent adoption of the dog or recent inclusion in the RUSVM teaching colony. Of the total dogs examined, and using the most recent sample results, the positivity rate for *Ancylostoma* was 9.9% (23 of 232) (Table 2). The *Ancylostoma* positivity rate was highest in the 6- to 12-month-old dogs (5 of 27; 18.5%). Most of the dogs (121 of 232; 52.2%) were on a monthly heartworm disease preventative [dogs receiving Revolution® (selamectin) were excluded in this count, as it is not registered for the control or treatment of *Ancylostoma*)]. For 32.3% (75 of 232) of the dogs, no anthelmintics had been dispensed by the clinic within the previous 6 months. However, not all dog owners acquire anthelmintics through the clinic; hence, anthelmintic use in these dogs cannot be ruled out. Positivity rate was lowest in the dogs for which prescription of a monthly preventative was recorded in the database. Of the 40 dogs with repeat fecal submissions, 47.5% (19 of 40) had a positive first sample result, and all were negative on second sample examination.

**Fig. 2:**
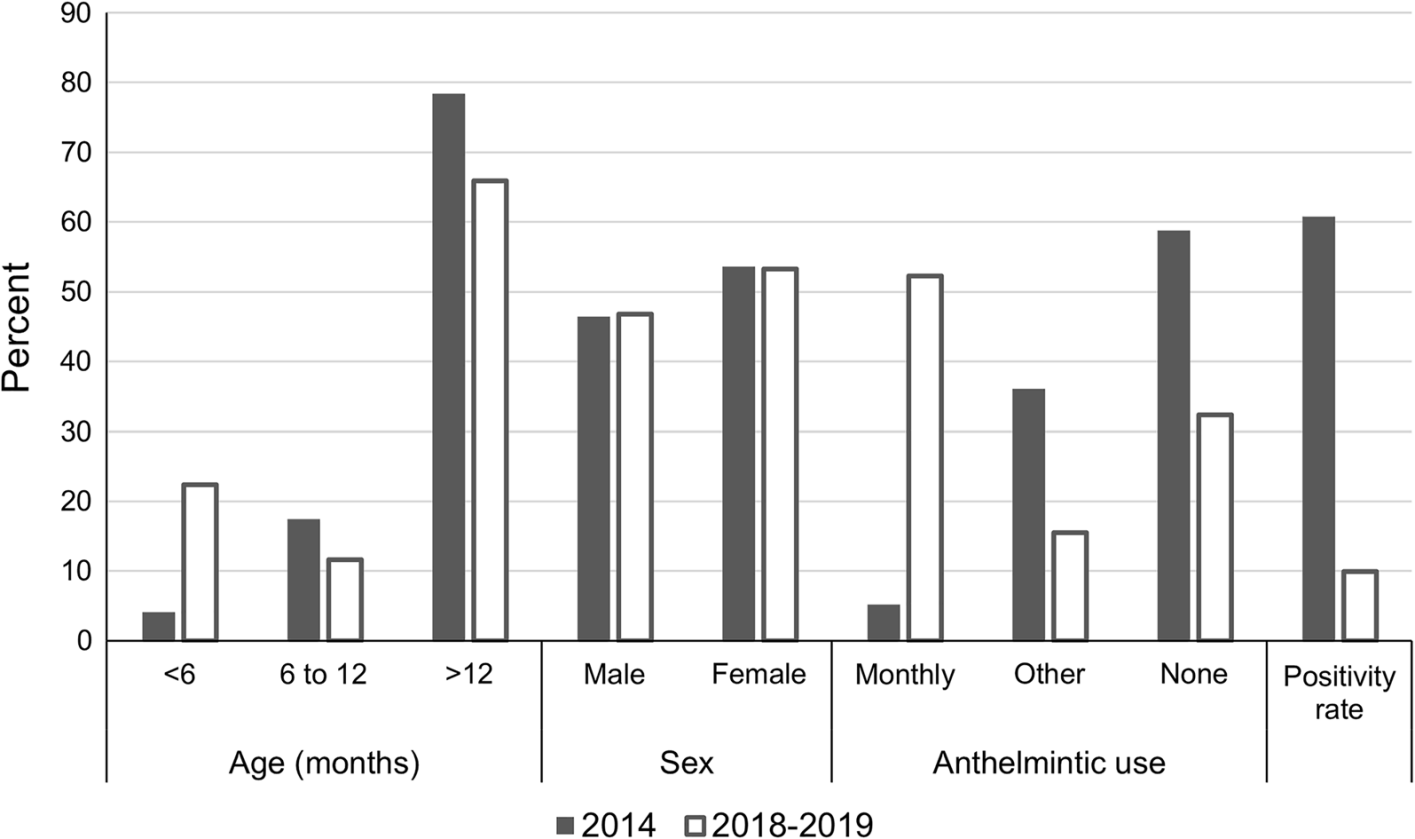
Descriptive characteristics and *Ancylostoma* positivity rate in two dog populations on St. Kitts. The 2014 data are from Kittitian owned dogs representing dogs with limited veterinary care. The 2018–2019 data represent dogs primarily owned by Ross University School of Veterinary Medicine or students attending the university

Within each dog population, anthelmintic use was the only statistically significant factor regarding the *Ancylostoma* positivity rate (Fisher’s exact test, *P* = 0.0299 for 2014 and *P* = 0.0154 for 2018–2019). Neither age nor gender had a significant effect on *Ancylostoma* positivity rate. In comparing the data sets, the 2014 and 2018–2019 dogs were different in age (Fisher’s exact test, *P* = 0.0002) with the 2018–2019 data having more < 6-month-old dogs and fewer > 12-month-old dogs, but the same sex ratio (chi-square test, *χ*^2^ = 0, *df* = 1, *P* = 1.0). Anthelmintic use was significantly different (Fisher’s exact test, *P* < 0.0001) with more use in the 2018–2019 population, and the overall positivity rate was different (chi-square test, *χ*^2^ = 92.05, *df* = 1, *P* < 0.0001) with a lower rate in the 2018–2019 population (Table 2; Fig. 2).

## Discussion

Comparing the available data across islands and over time is challenging given the different methods for diagnosis used and their related sensitivities. The “gold standard," necropsy, was used primarily in older studies with the remaining studies relying on microscopic examination of fecal samples. Although fecal examination through microscopy is the most widely used diagnostic technique for detecting hookworm infections, there are inherent limitations. Some eggs seen could be from coprophagia, resulting in an overestimation of prevalence [8]. On the other hand, lack of ability to detect pre-patent and single-sex infections could result in an underestimation of prevalence [9]. In addition, simple flotation methods could result in lower detection of hookworm eggs compared to double centrifugation methods [6, 7]. In the case of the St. Kitts’ data, both flotation solutions had a high enough spg to float hookworm eggs; however, the addition of a 10-min wait prior to reading the samples could have increased detection of eggs for the 2014 population, making direct comparisons challenging [6]. Regardless of these limitations, and despite the limited data available for the region, it is clear that hookworm infections are common in dogs in the Caribbean, and there is no evidence that prevalence has changed over time.

The *Ancylostoma* prevalence in dogs has been more extensively studied and reported in other parts of the Americas, especially North America. The positivity rate on St. Kitts and the other islands is similar to what has been found in shelter dogs in the southeastern USA (54%) [10], but much higher than found in pet dogs in the southern USA (4%) [11]. Compared to available data from Mexico and Central America, where prevalence ranges from 13% to > 75% are reported, the positivity rate in the Caribbean is similar [12–15]. These regions face some of the same challenges as the Caribbean islands regarding free-roaming dogs, related to the lack of environmental sanitation and climatic regions conducive to *Ancylostoma* environmental larval development and persistence.

In the data gathered here, ownership, age, and residence in a rural or urban area did not appear to significantly impact positivity rates in the dog populations sampled. Even within a well-cared-for pet population where year-round monthly broad-spectrum anthelmintics are used, positivity rate was 5.0%. The positivity rate in adult dogs and well-cared-for dogs suggests that there is a high environmental exposure. Most of the Caribbean islands have free-roaming dog populations which could lead to environmental contamination with feces and hence high exposure and reinfection rates [16–20].

Dogs develop only limited immunity to infection with *A. caninum*. Therefore, puppies and adult dogs contribute to environmental contamination with *Ancylostoma*, and the parasite may have impacts on the health of animals of any age. Thus, anthelmintic treatment more focused on puppies than adult dogs, as is the case in the Caribbean based on studies by El Amrani [21] and Morrison [22], may not be sufficient for *Ancylostoma* control. The impact of treatments focused on puppies with less frequent treatment of adult dogs is apparent in the two sets of data from St. Kitts. The positivity rate was lower in adult dogs seen at the veterinary clinic, which were typically on monthly treatments, compared to the adult dogs in the field study, which were rarely treated. This suggests that without veterinary care and guidance, owners may not be aware of the frequency in which treatment is required for adequate parasite control in adult dogs. Cost of monthly broad-spectrum anthelmintics can be prohibitive for many dog owners, given the general economy of the islands. Education and assistance in affording treatment are needed to decrease positivity, improve dog health, and decrease zoonotic risk from *Ancylostoma*. Increased use of monthly broad-spectrum anthelmintics would also have the benefit of providing protection against *Dirofilaria immitis*, which is endemic in much of the region.

A major knowledge gap evidenced in the data available is the prevalence of *A. caninum* versus *A. braziliense*, with the latter being less pathogenic in dogs but more likely to be associated with zoonotic infections. Only one of the published studies clearly differentiated the two species. Thus, a more accurate estimate of the true risk of zoonotic transmission cannot be determined from the data available. Also, dogs were the focus for this review and study of *Ancylostoma*. However, other animals, such as cats, may also become infected with *Ancylostoma* species that can be of zoonotic concern, particularly *A. braziliense*. Hence, further research, not only to garner more recent data for canine infections, to elucidate the prevalence of each of the *Ancylostoma* species, but also to understand the role of other animals in contributing to environmental exposure and zoonotic risk, is needed within the region.

## Conclusion

This review of Caribbean literature and data from St. Kitts sought to obtain a better understanding of the prevalence of *Ancylostoma* in dogs in the region. The results suggest a relatively high prevalence on St. Kitts, which is consistent with the results of the literature review for the broader Caribbean region. The lack of obvious change in prevalence over time within the region indicates a need for dog-owner education and increased anthelmintic use with a focus on adult dogs and not just puppies. Changes in dog management with fewer free-roaming dogs and more sanitary measures (i.e., picking up and disposal of feces) also could have significant impacts.

## Supplementary Information

**Additional file 1: Table S1**. Ancylostoma positivity rate by Parish for St. Kitts, 2014.

## Data Availability

Raw data for the 2014 field study and the 2018–2019 database are available on request.

## Abbreviations

Epg: Eggs per gram
IACUC: Institutional Animal Care and Use Committee
IRB: Internal Review Board
RUSVM: Ross University School of Veterinary Medicine
spg: Specific gravity
