# Supplementary material for: Ancylostoma in dogs in the Caribbean: a review and study from St. Kitts, West Indies
Source: Parasit Vectors. 2022 Apr 21;15:139. doi: 10.1186/s13071-022-05254-2 (PMC9027451; doi:10.1186/s13071-022-05254-2)
Supplement: Supplementary file 1 — Additional file 1: Table S1. Ancylostoma positivity rate by Parish for St. Kitts, 2014. [file 13071_2022_5254_MOESM1_ESM.docx]

**Additional file 1: Table S1.** *Ancylostoma* positivity rate by Parish for St. Kitts, 2014.

| Parish | Number tested^b^ | Sex | Age (months) | Number positive (%) |
| --- | --- | --- | --- | --- |
| Trinity Palmetto Point^a^ | 12 | 5 M; 7 F | 6: 6-12  6: >12 | 8 (66.7) |
| Saint Thomas Middle Island | 11 | 6M; 5 F | 1: <6  1: 6-12  9: >12 | 8 (72.7) |
| Saint Anne Sandy Point | 9 | 5 M; 4 F | 3: 6-12  6: >12 | 3 (33.3) |
| Saint Paul Capisterre | 10 | 4 M; 6 F | 3: 6-12  7: >12 | 8 (80.0) |
| Saint John Capisterre | 12 | 5 M; 7 F | 2: 6-12  10: >12 | 9 (75.0) |
| Christ Church Nichola Town | 8 | 3 M; 5 F | All: >12 | 6 (75.0) |
| Saint Mary Cayon | 11 | 5 M; 6 F | 1: <6  1: 6-12  9: >12 | 3 (27.2) |
| Saint Peter Basseterre^a^ | 9 | 6 M; 3 F | 1: 6-12  8: >12 | 5 (55.6) |
| Saint George Basseterre^a^ | 15 | 6 M; 9 F | 2: <6  13: >12 | 9 (60.0) |
| **Total** | **97** | **45 M; 52 F** | **4: <6**  **17: 6-12**  **76: >12** | **59 (60.8)** |

For comparative purposes, the positivity rate from 2018-2019 clinic data from primarily the Ross University School of Veterinary Medicine (RUSVM) kennels and RUSVM students 9.9% (23 of 232 dogs).

^a^ Parishes in which most RUSVM students reside; RUSVM is located in Trinity Palmetto Point. However, many dogs were adopted from the island and their Parish of origin is not known.

^b^ The aim was 10-12 dogs per Parish. However, in some Parishes insufficient feces for analysis were collected from the dogs. Additional dogs were sampled from Saint George Basseterre, the most populated of the nine Parishes.
